# Searching for the Molecular Basis of Partial Deafness

**DOI:** 10.3390/ijms23116029

**Published:** 2022-05-27

**Authors:** Dominika Oziębło, Natalia Bałdyga, Marcin L. Leja, Henryk Skarżyński, Monika Ołdak

**Affiliations:** 1 Department of Genetics, Institute of Physiology and Pathology of Hearing, 02-042 Warsaw, Poland; d.ozieblo@ifps.org.pl (D.O.); n.baldyga@ifps.org.pl (N.B.); m.leja@ifps.org.pl (M.L.L.); 2 Postgraduate School of Molecular Medicine, Medical University of Warsaw, 02-091 Warsaw, Poland; 3 Oto-Rhino-Laryngology Surgery Clinic, Institute of Physiology and Pathology of Hearing, 02-042 Warsaw, Poland; h.skarzynski@ifps.org.pl

**Keywords:** cochlear implantation, genetics, high-throughput sequencing, hearing loss, partial deafness, PDT-EC, PDT-EAS, gene, pathogenic variant

## Abstract

Hearing is an important human sense for communicating and connecting with others. Partial deafness (PD) is a common hearing problem, in which there is a down-sloping audiogram. In this study, we apply a practical system for classifying PD patients, used for treatment purposes, to distinguish two groups of patients: one with almost normal hearing thresholds at low frequencies (PDT-EC, *n* = 20), and a second group with poorer thresholds at those same low frequencies (PDT-EAS, *n* = 20). After performing comprehensive genetic testing with a panel of 237 genes, we found that genetic factors can explain a significant proportion of both PDT-EC and PDT-EAS hearing losses, accounting, respectively, for approx. one-fifth and one-half of all the cases in our cohort. Most of the causative variants were located in dominant and recessive genes previously linked to PD, but more than half of the variants were novel. Among the contributors to PDT-EC we identified *OSBPL2* and *SYNE4*, two relatively new hereditary hearing loss genes with a low publication profile. Our study revealed that, for all PD patients, a postlingual hearing loss more severe in the low-frequency range is associated with a higher detection rate of causative variants. Isolating a genetic cause of PD is important in terms of prognosis, therapeutic effectiveness, and risk of recurrence.

## 1. Introduction

The ability to detect, localize, and identify sounds is important for speech development, connecting and communicating with others, and orienting in the environment. Hearing sensitivity is assessed by pure-tone audiometry, which determines in decibels (dB) the magnitude by which the individual’s hearing deviates from the normal (0 dB) hearing level. In order to clearly understand speech, the ability to hear across the speech range of frequencies (125 Hz to 8 kHz) needs to be maintained. If patients have relatively good low-frequency hearing, but severe-to-profound hearing loss (HL) in the mid-to-high frequencies, the condition is defined as partial deafness (PD) [1,2].

To improve the hearing ability of PD patients, two major approaches are used. In acoustic stimulation (AS), the sounds entering the ear are amplified by hearing aids (HAs) or middle ear implants, methods which rely on the integrity and function of the inner ear. If the inner ear is damaged, the auditory nerve has to be stimulated directly, and this is done by electrical stimulation (ES) from a cochlear implant (CI). ES was first introduced for patients with severe-to-profound HL who experience limited or no benefit from a HA. With successful outcomes and growing expertise in cochlear implantation, the eligibility criteria for ES have been expanded [3,4,5].

ES is mainly intended for patients with down-sloping audiograms whose hearing impairment at high frequencies cannot be effectively compensated by a HA [6]. For this type of PD, appropriate treatment procedures have been developed (PDT). For those patients with normal (or slightly elevated) thresholds at low frequencies who have severe-to-profound HL at higher frequencies, electric complementation (EC) of natural low-frequency hearing has been proposed, designated as PDT-ENS or PDT-EC. On the other hand, for patients with only residual hearing at low frequencies and severe-to-profound HL at higher frequencies a combination of acoustic amplification of low-frequency hearing and electric stimulation in the same ear (PDT-EAS) has been introduced [2,7,8].

It is well-accepted that genetic factors play an important role in the development of HL. In this regard, the most studied form of HL is prelingual profound HL, where it has been estimated that about 50% of cases are due to genetic causes [9,10,11]. There are continuing efforts to elucidate the genetic background of deafness, and novel candidate genes have been identified. The leading causes of profound HL are recessive pathogenic variants at the DFNB1 locus (encompassing the *GJB2* and *GJB6* genes) [12,13]. In comparison, much less is known about the molecular basis of HL in those PD patients for whom the high frequencies are mainly affected. Here, we show the results of a comprehensive genetic testing of patients with PDT-EC (*n* = 20) and PDT-EAS (*n* = 20).

## 2. Results

### 2.1. Audiological Data

**Figure 1:**
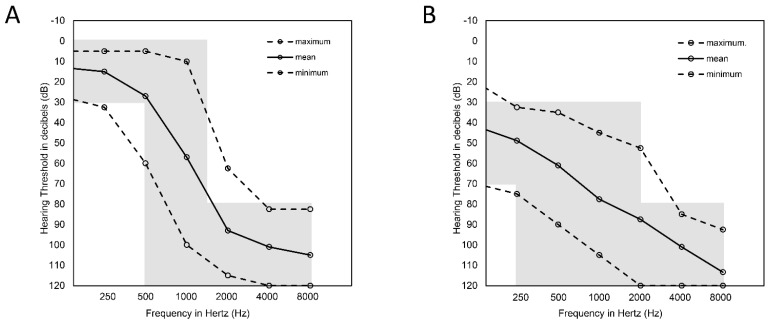
Mean pure-tone audiograms of the analyzed PDT patients. (**A**) Binaural hearing thresholds in the PDT-EC group; (**B**) Binaural hearing thresholds in the PDT-EAS group. Solid lines represent the average hearing level and dashed lines represent the maximum and minimum hearing thresholds.

In the PDT-EC group, patients had normal hearing (or only mild HL) at low frequencies, moderate HL at mid frequencies, and severe-to-profound HL at high frequencies (Figure 1A). The mean age at HL onset was 13 (from 3 to 35 years). In 45% (9/20) of PDT-EC patients, there was a familial form of HL. There were 7 patients who had pre- or perilingual HL, while 13 individuals had postlingual HL. In five patients, their HL was asymmetric, and in another four it was progressive. There were 10 patients who had been fitted with HAs only, 4 who had received both HAs and CIs, 4 who had CIs only, and 2 who had neither HAs nor CIs (Table 1).

In the PDT-EAS group, patients had moderate HL at low frequencies and severe-to-profound HL at mid and high frequencies (Figure 1B). The mean age at HL onset was 18 (from congenital to 50 years). In 50% (10/20) of the PDT-EAS patients, HL was also diagnosed in other family members. Pre- or perilingual HL was observed in 5 patients and postlingual HL was identified in 15 patients. All patients had symmetric HL and five of them had a progressive form of the disease. There were 12 patients who were fitted with HAs only, 4 received both HAs and CIs, 3 used only CIs, and 1 used neither HAs nor CIs (Table 1).

### 2.2. Genetic Results

Multigene panel testing of the probands’ DNA samples generated, on average, 3,102,233 reads for the PDT-EC group and 3,088,792 reads for the PDT-EAS group. The targeted region was mapped in 99.81% and 99.85%, respectively. The mean coverage for PDT-EC samples was 83.21× and for PDT-EAS 83.13×. In both groups, 97.37% and 98.21% of the targeted region was covered at least 20×. Sequencing metrics for particular samples are given in Table S1.

For every patient, pathogenic, likely pathogenic, and variants of unknown significance (VUS) were selected (Table S2) and further analyzed in the context of literature data and the results of segregation analyses. Probably causative variants were detected in 35% (14/40) of the tested families (Table 2). They were identified in as many as 25% (3/12) of patients with pre- or perilingual HL and 39% (11/28) of patients with postlingual HL.

#### 2.2.1. Genetic Background of HL in the PDT-EC Group

Genetic testing revealed probably causative variants in 20% (4/20) of families from the PDT-EC group. The majority of them were novel (83%, 5/6) and not previously linked with HL. Identified probably causative variants were located in the *CDH23*, *KCNQ4*, *OSBPL2,* and *SYNE4* genes.

**Figure 2:**
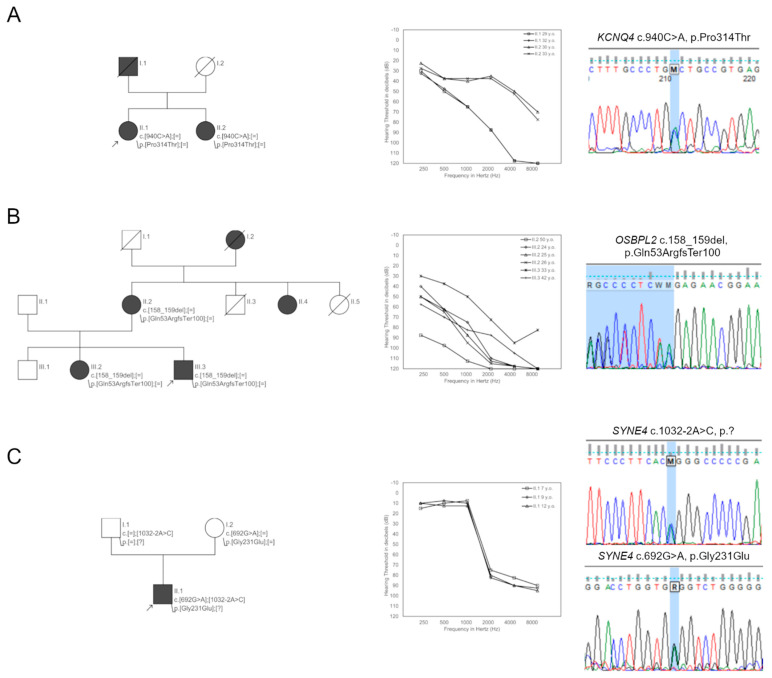
Selected genetic and audiometric data of PDT-EC patients. Families with causative variants in the *KCNQ4* gene (**A**), in the *OSBPL2* gene (**B**), and in the *SYNE4* gene (**C**).

In Families 9 and 12, heterozygous variants leading to autosomal dominant HL (ADHL) were identified. In the proband from Family 9, the age of HL onset was 14 years. After genetic testing, a heterozygous transversion c.940C>A (p.Pro314Thr) in the *KCNQ4* gene was identified. This variant is absent in population databases and in silico pathogenicity prediction tools reveal its deleterious role. Segregation studies confirmed the presence of c.940C>A variant in the proband’s sister, who was also diagnosed with HL (Figure 2A).

In the proband from Family 12, HL was first diagnosed at age 12. A heterozygous c.158_159del (p.Gln53ArgfsTer100) variant in the *OSBPL2* gene was identified (Figure 2B). This variant truncates the protein and is not recorded in population databases. In 2019, it was identified as causative for HL in a Mongolian family [14] and included in the HGMD database (CD193739).

In Families 14 and 17, compound heterozygous variants causative for autosomal recessive HL (ARHL) were selected. The proband from Family 14 was diagnosed with HL at the age of 20 and high-throughput sequencing resulted in the identification of c.2005C>T (p.Pro669Ser) and c.2864G>T (p.Arg955Leu) variants in the *CDH23* gene. These variants are novel, have only sporadically been reported in population databases, and are predicted as pathogenic by the majority of applied algorithms. Unfortunately, genetic material for segregation analysis was not available from the family members.

In Family 17, the proband had HL from the age of 6. In this patient, c.692G>A (p.Gly231Glu) and c.1032-2A>C (p.?) variants in the *SYNE4* gene were found. The c.692G>A has a very low allele frequency and the c.1032-2A>C change is absent in population databases. Both variants are predicted to affect transcript processing. Family studies confirmed an *in trans* configuration of the identified *SYNE4* variants (Figure 2C).

#### 2.2.2. Genetic Background of HL in the PDT-EAS Group

**Figure 3:**
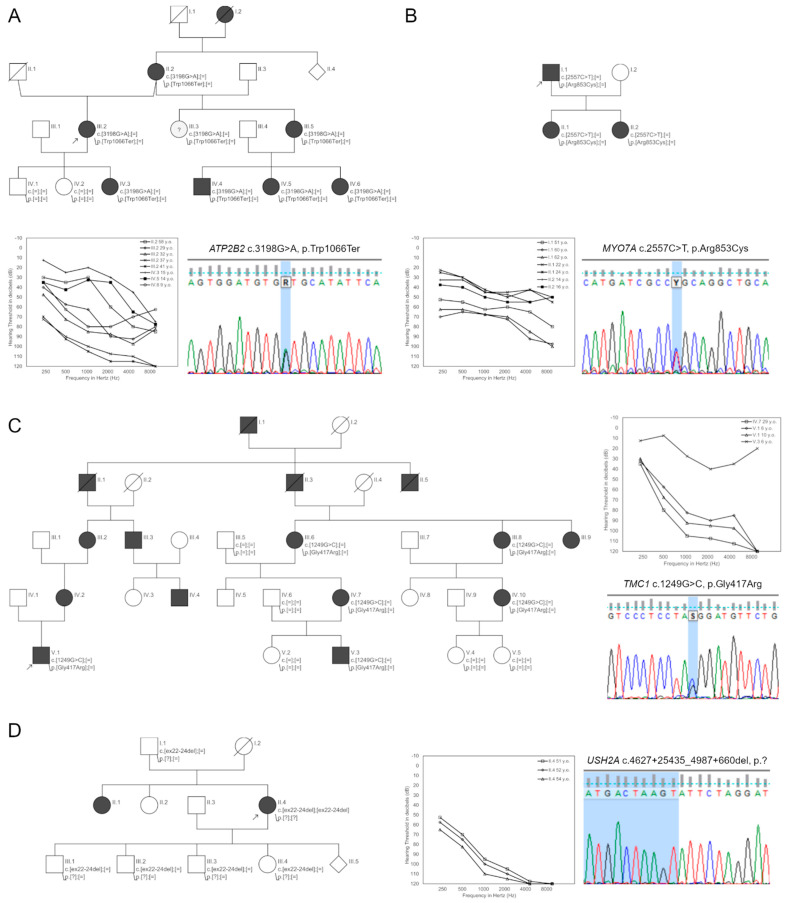
Selected genetic and audiometric data of PDT-EAS patients. Families with causative variants in the *ATP2B2* gene (**A**), in the *MYO7A* gene (**B**), in the *TMC1* gene (**C**), and in the *USH2A* gene (**D**).

Probably causative variants were detected in 50% (10/20) of patients from the PDT-EAS group and 50% (7/14) of them were novel. They were located in the *ATP2B2*, *LOXHD1*, *MYO6*, *MYO7A*, *PTPN11*, *TMC1*, *TMPRSS3,* and *TRIOBP* genes. In Families 24, 34, 35, 37, and 38 heterozygous probably causative variants for ADHL were identified. The proband from Family 24 was diagnosed with progressive HL at the age of 15. She carried a novel c.3198G>A (p.Trp1066Ter) variant in the *ATP2B2* gene. This stop-gained genetic alteration is absent in population databases, and segregation studies confirmed its presence in six other individuals with HL in this family (Figure 3A).

In Family 34, a c.1226G>C (p.Gly409Ala) variant in the *PTPN11* gene was identified as causative for HL diagnosed at age 45. This missense change has a low frequency in population databases and has previously been included in HGMD as causative of a mild form of Noonan syndrome (CM070248). Based on segregation studies, the c.3198G>A variant was inherited from the patient’s mother, but her hearing status is unknown.

In Family 35, the proband was diagnosed with progressive HL at the age of 50. Genetic testing detected a c.2557C>T (p.Arg853Cys) variant in the *MYO7A* gene (Figure 3B). It is absent in population databases and predicted to be pathogenic. In 2004, this variant was also identified in a German family. It has been functionally validated [15] and reported in HGMD (CM042433).

In Family 37, a novel c.1417A>G (p.Ile473Val) variant in the *MYO6* gene was found. Based on population frequencies and pathogenicity predictions, it was identified as probably causative for HL; however, there were no available DNA samples for family studies.

The proband from Family 38 had HL from the age of 3. After high-throughput sequencing, a c.1249G>C (p.Gly417Arg) variant in the *TMC1* gene was chosen for further family studies. This variant is absent in population databases and predicted to be pathogenic. The c.1249G>C variant was present in six family members with progressive HL (Figure 3C).

In Families 21, 25, 30, 33, and 36 compound heterozygous variants causative for ARHL were selected. The proband from Family 21 carries c.1919T>C (p.Leu640Pro) and c.2030T>C (p.Ile677Thr) variants in the *TMC1* gene. The c.1919T>C variant has never been described in the context of HL development and is not present in population databases. All pathogenicity predictors suggest its causative role. The second variant, c.2030T>, is a known ARHL-causing mutation (HGMD CM094820).

In the proband from Family 25, HL was diagnosed during newborn hearing screening. Two novel c.1228C>T (p.Arg859Trp) and c.2575C>T (p.Gln410Ter) variants in the *LOXHD1* gene were selected as probably causative for this phenotype. The first c.1228C>T variant has low allele frequency in population databases and the majority of in silico algorithms predict its pathogenicity. The c.2575C>T variant terminates the protein and is absent in population databases.

The proband from Family 33 has had HL from the age of 15. In multigene panel testing, two known pathogenic variants, c.1276G>A (p.Ala426Thr) and c.1343T>C (p.Met448Thr), were identified in *TMPRSS3*. Both have low allele frequencies in population databases, strong pathogenicity predictions, and are present in HGMD (CM116227, CM179141).

DNA samples from family members were available for segregation studies in Families 30 and 36 only. In these families, HL was diagnosed at the ages of 6 and 17, respectively. In addition to HL, the proband from Family 30 also suffers from retinitis pigmentosa, and genetic testing identified a homozygous deletion encompassing exons 22–24 of the *USH2A* gene (Figure 3D). In Family 36, truncating c.3004del (p.Ala1002LeufsTer3) and c.5014G>T (p.Gly1672Ter) variants in the *TRIOBP* gene were selected. These nucleotide changes are absent or very rare in population databases and family studies confirmed their *in trans* configuration.

## 3. Discussion

In this study, we have analyzed the genetic background of HL in two groups of patients with PD, both of which had strongly elevated thresholds for high-frequency sounds. We used pure-tone audiometry data to assign the patients into one of two groups: PDT-EC, which had steep down-sloping audiograms, and PDT-EAS, in which the slope was gentler (Figure 1). For those patients for whom we achieved a genetic diagnosis, we sought to assess the evolution of HL based on the audiometric data collected from different time points and from available family members. *ATP2B2, TMC1,* and *MYO7A* were among the genes that attracted our attention. All these genes caused ADHL which progressed over time, although they showed an intrafamilial variability in terms of the degree of HL. This observation has strong practical implications for patients undergoing cochlear implantation. If the results of genetic testing are available, clinicians and patients will then be aware of the risk of losing residual hearing as a result of disease progression. This knowledge could lead to better patient care in terms of selecting an appropriate electrical stimulation method and rehabilitation program.

In probands from both patient groups, we performed a multigene panel containing most of the known isolated HL genes and a majority of syndromic HL genes. We excluded patients with pathogenic variants at the DFNB1 locus (*GJB2* and *GJB6* genes) and the mitochondrial m.1555A>G variant in the *MT-RNR1* gene, which are routinely tested in our diagnostic scheme. Although the majority of DFNB1 variants result in profound HL affecting all frequencies almost uniformly, some *GJB2* variants may also contribute to typical audiometric curves of PDT-EC and PDT-EAS [16,17]. Similarly, m.1555A>G may also lead to both (PDT-EC and PDT-EAS) HL phenotypes [18].

Using stringent criteria from the ACMG guidelines, we classified the identified genetic variants and selected only those assigned as being pathogenic, likely pathogenic, or variants of unknown significance (VUS). After combining the available published data with characteristic HL phenotypes, we chose probably causative variants from this group of mutations. Variants considered as VUS will need to be reevaluated in the future. This may require the participation of family members (to establish the configuration of the identified variants), identification of novel HL cases, or functional studies. On average, our causative variant detection rate was 35% (14/40), which matches the value of approx. 34% for down-sloping HL in patients of Asian origin [19,20,21]. We were unable to verify this finding with European patients. Interestingly, the genetic diagnostic rates varied between the PDT-EC (20%, 4/20) and PDT-EAS (50%, 10/20) groups, indicating that the greater the degree of HL, the more likely it is to find a genetic cause.

When we examined the age of HL onset, a higher diagnostic rate was obtained for patients with postlingual HL (39%, 11/28) than for patients with pre- and perilingual HL (25%, 3/12). In PDT-EC patients with pre- or perilingual onset of HL, we could not identify any causative variants. A similar observation was reported by Rim et al., who found that, in patients with early HL onset, the ski-slope HL group showed a lower probability of genetic diagnosis [20]. This is an interesting and surprising finding as one might expect that a range of environmental factors contribute to postlingual HL development.

To our knowledge, the sequencing strategy applied here covered all genes reported to cause ski-slope HL—except for the *DLL1* gene, which has recently been proposed as a candidate gene causing this type of HL [20]. Thus, we were unable to determine the role of *DLL1* in the development of HL in our patients, although we do have plans to sequence this gene in those individuals who do not have a molecular diagnosis. In this study, we have analyzed protein coding, gene splice-site regions, and copy number variants (CNVs). Our approach does not reliably detect CNVs in genes having pseudogenes. It is especially relevant for *STRC* and *OTOA* genes, but these genes are usually involved in milder HL phenotypes [22,23]. It is notable that, for some of our patients, we detected single causative variants in recessive HL genes but the second variant was missing. Since there is a characteristic audiometric curve for some of these genes, it is tempting to speculate that the second causative variant may be located in non-coding regions (e.g., deep intronic or regulatory regions) [24]. One should also keep in mind that the patient may only be a carrier of the recessive variant and that their HL is a consequence of other genetic or environmental factors.

*TMPRSS3* has been repeatedly associated with a down-sloping audiogram. Depending on the pathogenic potential of the *TMPRSS3* mutations, the phenotypes can be divided into prelingual, profound HL (DFNB8), and postlingual-onset HL (with a ski-slope audiogram and a variable age of onset and progression rate—DFNB10). The pure-tone audiometric profiles of DFNB10 may fit into the PDT-EC or PDT-EAS HL spectrum [21,25,26]. The single patient in this study with DFNB10-related HL was classified as having PDT-EAS because of having two known *TMPRSS3* pathogenic variants [25,27,28]. In the available literature, we could not find any patient with the same *TMPRSS3* variant combination, but after searching our database we did identify another individual with the same *TMPRSS3* variant composition and audiogram corresponding to PDT-EC (data not shown).

We also detected causative variants in a set of other genes which have previously been related to down-sloping HL. This includes *KCNQ4*, *ATP2B2*, *PTPN11*, *MYO7A*, *MYO6*, and *TMC1* (causing ADHL) and *CDH23*, *TMC1*, *LOXHD1*, *TRIOBP,* and *USH2A* (that lead to ARHL) [19,20,29,30,31,32,33,34]. A nonobvious result to emerge from the data is that, in our cohort, *OSBPL2* and *SYNE4* also contribute to PDT-EC. Both of them are relatively new and uncommon players in the field of hereditary HL. For *OSBPL2*, only three pathogenic variants in five ADHL families have thus far been detected [14,35,36,37]. One of these variants was found in the patient described in this study. For *SYNE4,* two pathogenic variants (both in a homozygous state) have been reported: in two Iraqi Jewish families and one Turkish consanguineous family [38,39]. Here, we have found two novel *SYNE4* mutations in a compound heterozygous state which are predicted to affect pre-mRNA splicing and give the characteristic pattern of HL similar to that reported previously.

## 4. Materials and Methods

### 4.1. Study Subjects

A group of 40 patients who met audiological criteria of PDT was recruited. Based on pure-tone audiometry (PTA) data, they were divided into two subgroups: PDT-EC (*n* = 20) with mean binaural hearing thresholds 125 Hz ≤ 30 dB, 250 Hz ≤ 30 dB, 500 and 1000 Hz from 0 to 120 dB, 2000 Hz ≥ 80 dB, 4000 Hz ≥ 80 dB, 8000 Hz ≥ 80 dB; and PDT-EAS (*n* = 20) with mean binaural hearing thresholds 125 Hz ≥ 30 and ≤ 70 dB, 250 Hz ≥ 30, 500 Hz ≥ 30, 1000 Hz ≥ 30, 2000 Hz ≥ 30, 4000 Hz ≥ 80 dB, 8000 Hz ≥ 80 dB. The PTA results of CI ears were excluded to examine only the natural course of the disease. In patients with asymmetric HL, PTA data from the ear with better hearing were excluded.

Each group consisted of 13 females and 7 males. Prior to recruitment, all patients were prescreened for pathogenic variants in the DFNB1 locus and for the m.1555A>G variant in the mitochondrial genome. They had no environmental risk factors for HL development. Written informed consent was obtained from participants or their legal guardians. The study was approved by the ethics committee at the Institute of Physiology and Pathology of Hearing (KB.IFPS.25/2017, KB.IFPS:26/2020) and performed according to the Declaration of Helsinki.

### 4.2. Analysis of the Audiological Data

All available PTA results for the probands were analyzed (Figure S1). Asymmetric HL was recognized when there was a difference of ≥15 dB between the right and left ears at three contiguous frequencies. In patients who had PTA results at least 3 years apart, the progression of HL was assessed and it was defined as progressive if there was a change in hearing thresholds of ≥20 dB at two or more adjacent frequencies. Additionally, based on the patients’ family history, they were described as sporadic HL cases (only one occurrence of HL in the family) or familial (at least two individuals with a similar type of HL).

### 4.3. Genetic Testing

Whole blood samples were collected from probands and genomic DNA was isolated using the standard salting-out procedure. From available family members, buccal swabs were obtained and genomic DNA was isolated with the automatic method on a Maxwell RSC Instrument (Promega, Germany) according to the manufacturer’s protocol. A custom multigene panel test containing 237 HL-related genes (Table S3) (SeqCap EZ Choice Probes, Roche, Switzerland) was performed on proband samples. The quantity and quality of genomic DNA were evaluated and the material was further used in the library preparation process. Enriched libraries were pooled and sequenced using the MiSeq Reagent Kit V3 (150 cycles, 2 × 75 bp) and the MiSeq Instrument (Illumina, San Diego, CA, USA).

Bioinformatic analyses of the obtained data were performed as described previously [40]. Additionally, CNVs were analyzed with the DECoN tool [41]. Separate approaches for CNVs detection in genes with pseudogenes (e.g., *STRC* and *OTOA* genes) were not applied. The pathogenicity of the identified variants was evaluated based on the ACMG/AMP Interpreting Sequence Guidelines [42] with further specifications for HL [43]. Classification criteria included: allele frequency (gnomAD, UK10K, ESP databases), in silico predictions (PolyPhen-2, SIFT, Mutation Taster, LRT, and CADD), annotations from public variant databases (ClinVar, HGMD), matches from an in-house variants database, and related medical literature.

The presence of probably causative variants (pathogenic, probably pathogenic, and VUS) was confirmed and segregation analyses were performed using standard Sanger sequencing.

## 5. Conclusions

Hereditary causes need to be taken into consideration when looking at the etiology of PD, both in familial and sporadic cases, particularly after ruling out the involvement of environmental causes of HL (such as TORCH, recurrent infections or trauma). Identifying the genetic causes of HL can provide important insights into the most appropriate personalized patient care. One practical aspect of genetic testing in PD may be its ability to predict the natural progression of HL and, consequently, the chance of preserving residual hearing after cochlear implantation.

## Figures and Tables

**Table 1:** Audiological characteristics of the PDT patients.

PDT-EC Group									PDT-EAS Group								
Family ID	Patient ID	Age at HL Onset (Years)	Age at Hearing Examination (Years)	HL			HAs	CI	Family ID	Patient ID	Age at HL Onset (Years)	Age at Hearing Examination (Years)	HL			HAs	CI
				Sporadic/Familial	Progression	Symmetry							Sporadic/Familial	Progression	Symmetry		
1	943	4	20	sporadic	−	+	+	−	21	1371	4	26	sporadic	−	+	−	+
2	1242	3	40	sporadic	−	−	+	−	22	4759	15	77	familial	+	+	+	+
3	3403	24	32	sporadic	−	+	−	+	23	4933	25	60	familial	−	+	+	−
4	5218	3	9	familial	−	+	+	+	24	5047	15	36	familial	+	+	−	+
5	6949	6	14	sporadic	−	+	+	+	25	8507	congenital	6	sporadic	−	+	+	+
6	7646	28	37	sporadic	+	+	−	+	26	9243	15	57	familial	−	+	+	−
7	8138	4	9	sporadic	−	+	−	+	27	9322	18	44	sporadic	−	+	+	−
8	8689	25	48	familial	N/A	+	+	−	28	9418	20	48	sporadic	N/A	+	+	−
9	9148	14	32	familial	−	+	+	−	29	9425	5	20	sporadic	N/A	+	+	−
10	9302	6	43	sporadic	N/A	−	+	−	30	9508	6	52	familial	N/A	+	+	+
11	9632	30	39	familial	−	+	−	−	31	9772	32	59	familial	−	+	−	+
12	9661	12	33	familial	+	+	+	+	32	10045	congenital	5	familial	N/A	+	+	−
13	9754	35	52	familial	+	+	−	+	33	10309	15	17	sporadic	N/A	+	−	−
14	9774	20	23	sporadic	N/A	−	+	−	34	10331	45	51	sporadic	N/A	+	+	−
15	9785	4	14	familial	+	+	+	−	35	10332	50	60	familial	+	+	+	−
16	9994	20	36	familial	N/A	+	+	−	36	10385	17	20	sporadic	+	+	+	−
17	10069	6	9	sporadic	−	+	+	−	37	10892	39	66	familial	N/A	+	+	−
18	11108	3	31	familial	−	−	+	−	38	11023	3	6	familial	−	+	+	−
19	13960	7	30	sporadic	−	−	−	−	39	11054	20	44	sporadic	−	+	+	+
20	14220	4	15	sporadic	N/A	+	+	+	40	11162	19	44	sporadic	+	+	+	−

HAs, hearing aids; CI, cochlear implant; ‘+’, present; ‘–’, absent.

**Table 2:** List of the identified probably causative variants.

Family ID	Proband ID	Gene	Variant cDNA Level	Variant Protein Level	Population Frequencies				Pathogenicity Predictions					ACMG Classification
					gnomAD	UK10K	EVS	SIFT	PolyPhen-2	Mutation Taster	LRT	CADD	SpliceAI	
Family9	9148	*KCNQ4*	**c.940C>A**	**p.(Pro314Thr)**	**0**	**0**	**0**	**D**	**D**	**D**	**D**	**29.9**	**–**	**VUS**
Family12	9661	*OSBPL2*	c.158_159del	p.(Gln53Argfs*100)	0	0	0	–	–	–	–	–	–	P
Family14	9774	*CDH23*	**c.2005C>T**	**p.(Pro669Ser)**	**0.000007**	**0**	**0**	**D**	**D**	**D**	**D**	**32**	**–**	**VUS**
			**c.2864G>T**	**p.(Arg955Leu)**	**0.00001**	**0.00001**	**0**		**D**	**D**	**D**	**27**	**–**	**LP**
Family17	10069	*SYNE4*	**c.1032-2A>C**	**p.(?)**	**0**	**0**	**0**	**–**	**–**	**D**	**–**	**19.6**	**A**	**P**
			**c.692G>A**	**p.(Gly231Glu)**	**0.0001**	**0**	**0**	**D**	**D**	**D**	**N**	**24.5**	**A**	**LP**
Family21	1371	*TMC1*	**c.1919T>C**	**p.(Leu640Pro)**	**0**	**0**	**0**	**D**	**D**	**D**	**D**	**29.5**	**–**	**VUS**
			c.2030T>C	p.(Ile677Thr)	0.00001	0	0	D	P	D	D	26.4	–	VUS
Family24	5074	*ATP2B2*	**c.3198G>A**	**p.(Trp1066Ter)**	**0**	**0**	**0**	**T**	**D**	**D**	**D**	**46**	**–**	**LP**
Family25	8507	*LOXHD1*	**c.2575C>T**	**p.(Arg859Trp)**	**0.0004**	**0**	**0.0002**	**D**	**D**	**D**	**D**	**14.8**	**–**	**VUS**
			**c.1228C>T**	**p.(Gln410Ter)**	**0**	**0**	**0**	**–**	**–**	**D**	**T**	**38**	**A**	**LP**
Family30	9508	*USH2A*	c.4627+25435_4987+660del	p.(?)	–	–	–	–	–	–	–	–	–	P
			c.4627+25435_4987+660del	p.(?)	–	–	–	–	–	–	–	–	–	P
Family33	10309	*TMPRSS3*	c.1343T>C	p.(Met448Thr)	0.00001	0	0	D	T	D	D	24.1	–	P
			c.1276G>A	p.(Ala426Thr)	0.001	0.0026	0.0014	D	D	D	D	29.7	–	P
Family34	10331	*PTPN11*	c.1226G>C	p.(Gly409Ala)	0.00002	0	0.00015	T	D	D	D	12.6	–	VUS
Family35	10332	*MYO7A*	c.2557C>T	p.(Arg853Cys)	0	0	0	T	D	D	D	34	–	P
Family36	10385	*TRIOBP*	**c.3004del**	p.(Ala1002Leufs*3)	**0**	**0**	**0**	**–**	**–**	**–**	**–**	**–**	**–**	**LP**
			c.5014G>T	p.(Gly1672Ter)	0.0004	0.0002	0.0003	–	–	D	–	37	–	P
Family37	10892	*MYO6*	**c.1417A>G**	**p.(Ile473Val)**	**0**	**0**	**0**	**D**	**D**	**D**	**D**	**23.7**	**–**	**VUS**
Family38	11023	*TMC1*	**c.1249G>C**	**p.(Gly417Arg)**	**0**	**0**	**0**	**D**	**D**	**D**	**D**	**23.7**	**–**	**LP**

Novel variants are bolded. P, pathogenic; LP, likely pathogenic; VUS, variant of unknown significance; D, deleterious; T, tolerated; A, affects; ‘–’, not applied. Reference sequences: *ATP2B2* NM_001001331.4 and NP_001001331.1, *CDH23* NM_022124.6 and NP_071407.4, *KCNQ4* NM_004700.4 and NP_004691.2, *LOXHD1* NM_001384474.1 and NP_001371403.1, *MYO6* NM_004999.4 and NP_004990.3, *MYO7A* NM_000260.4 and NP_000251.3, *OSBPL2* NM_144498.4 and NP_653081.1, *PTPN11* NM_002834.5 and NP_002825.3, *SYNE4* NM_001039876.3 and NP_001034965.1, *TMC1* NM_138691.3 and NP_619636.2, *TMPRSS3* NM_024022.4 and NP_076927.1, *TRIOBP* NM_001039141.3 and NP_001034230.1, *USH2A* NM_206933.4 and NP_996816.3.

## Data Availability

The data that support the findings of this study are available from the corresponding author upon request.

## Supplementary Materials

The following are available online at https://www.mdpi.com/article/10.3390/ijms23116029/s1.
