# Supplementary figures and images for: Searching for the Molecular Basis of Partial Deafness
Source: Int J Mol Sci. 2022 May 27;23(11):6029. doi: 10.3390/ijms23116029 (PMC9181477; doi:10.3390/ijms23116029)

Figure S1. PTA data of the genetically tested probands

PDT-EC

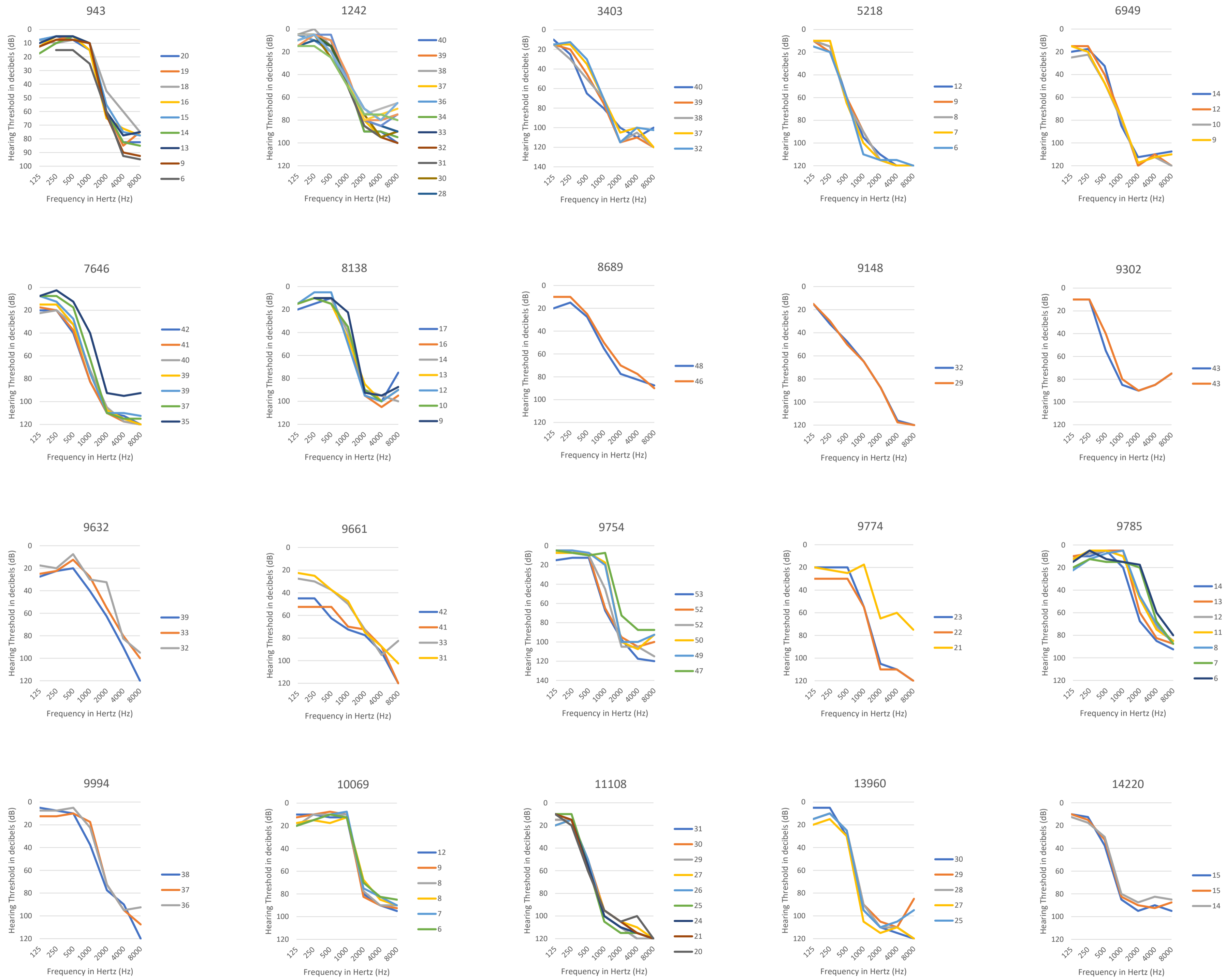

PDT-EAS

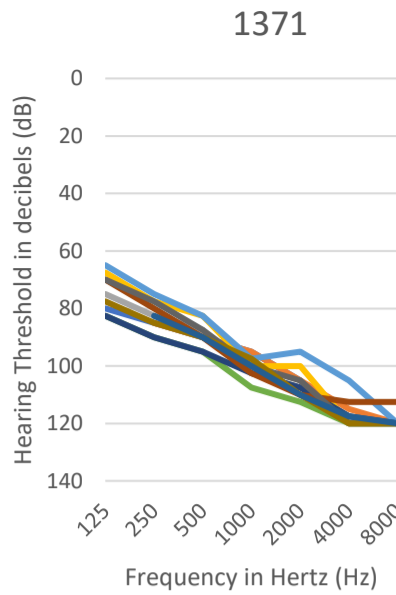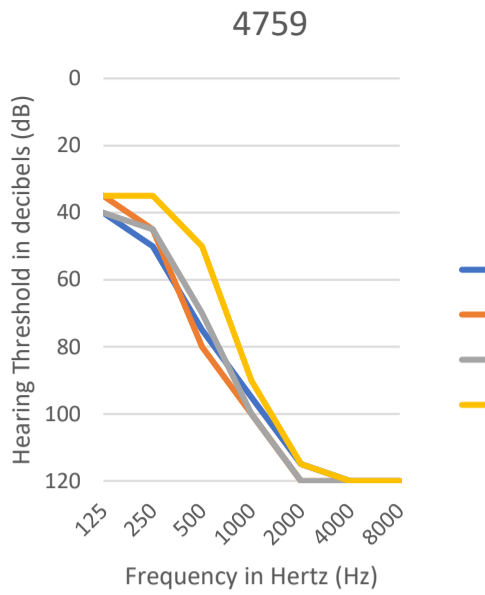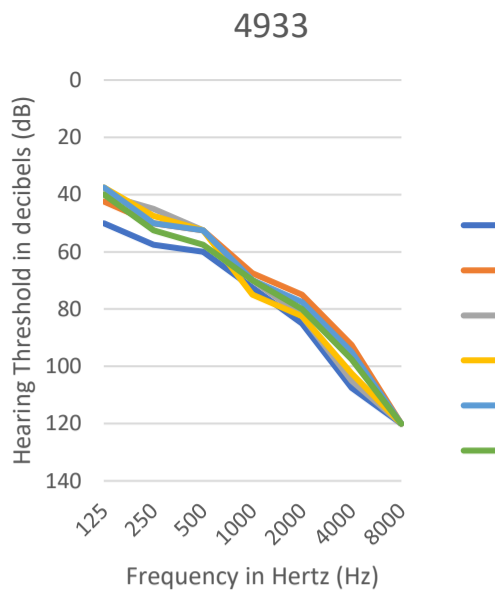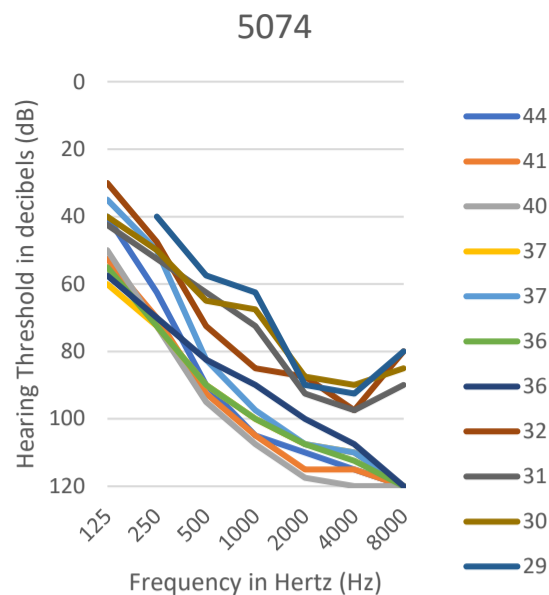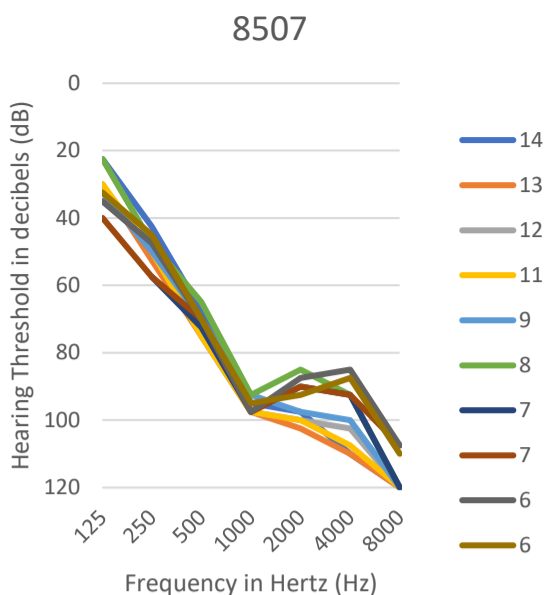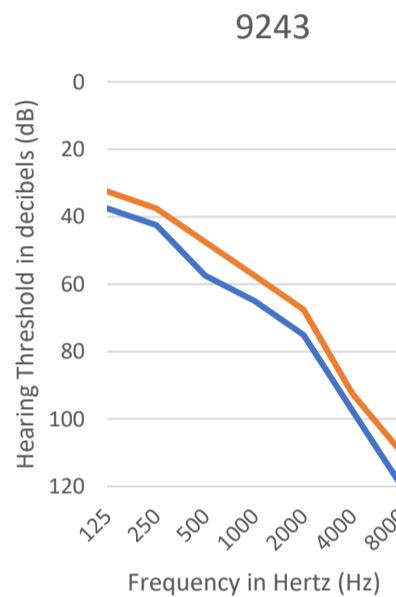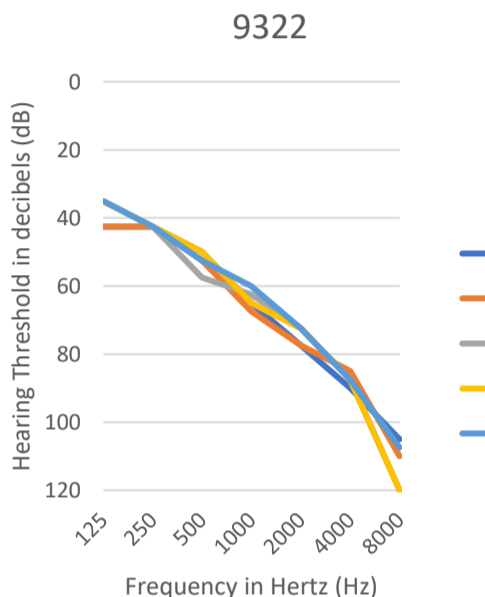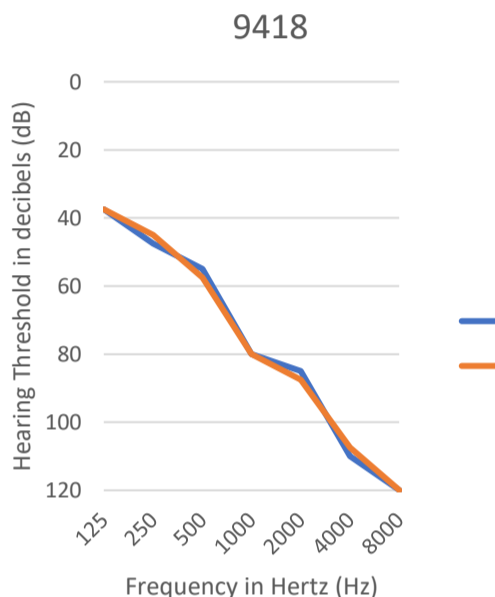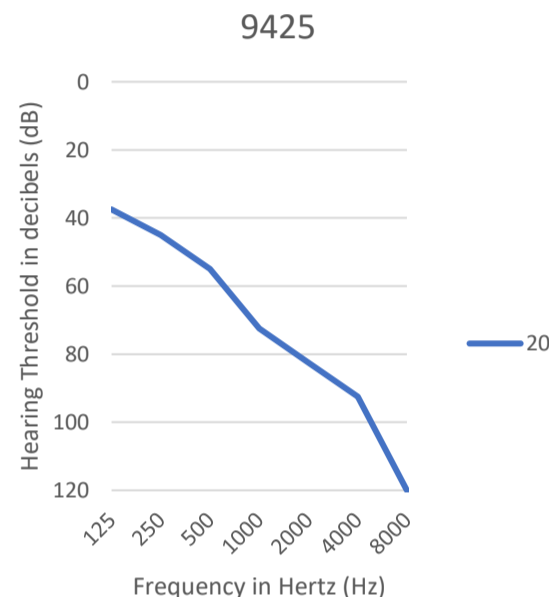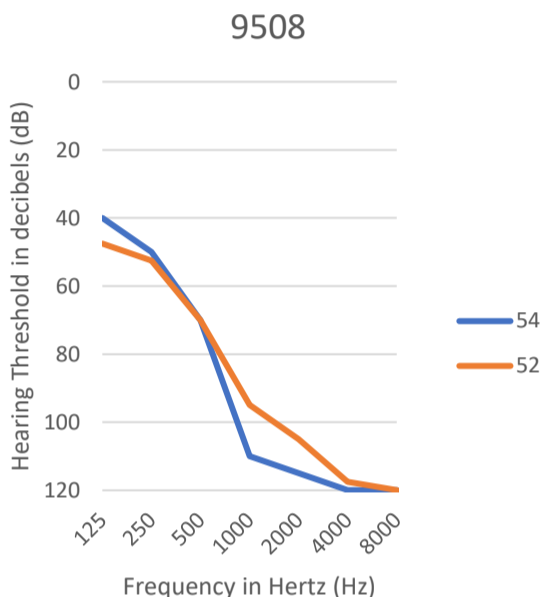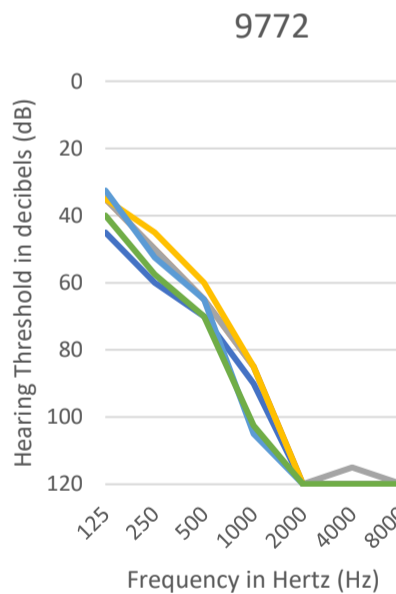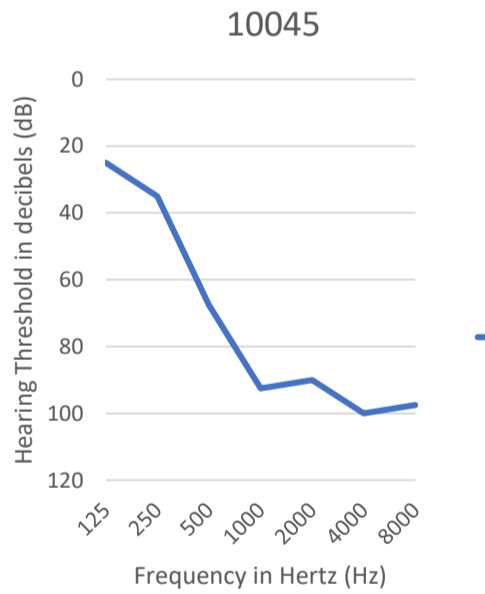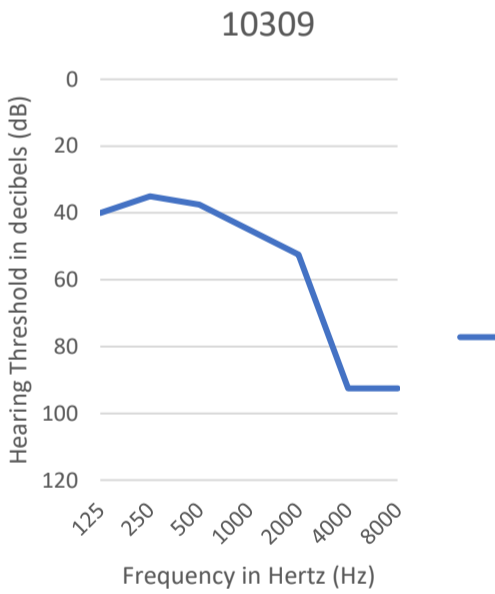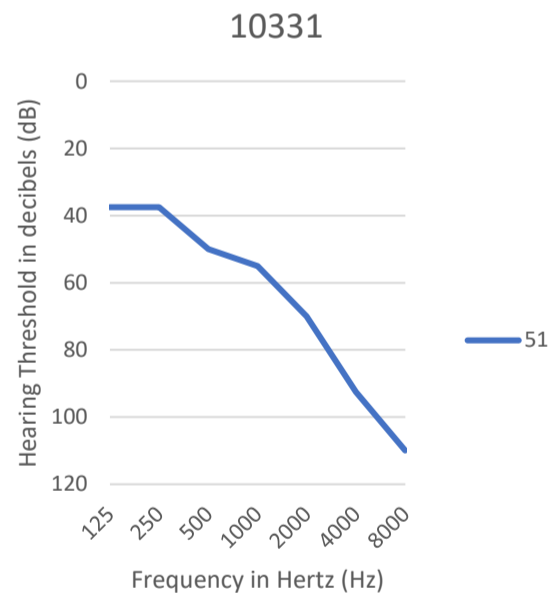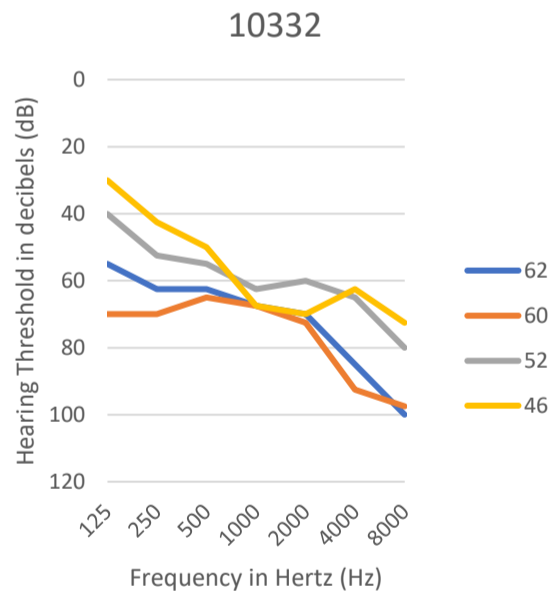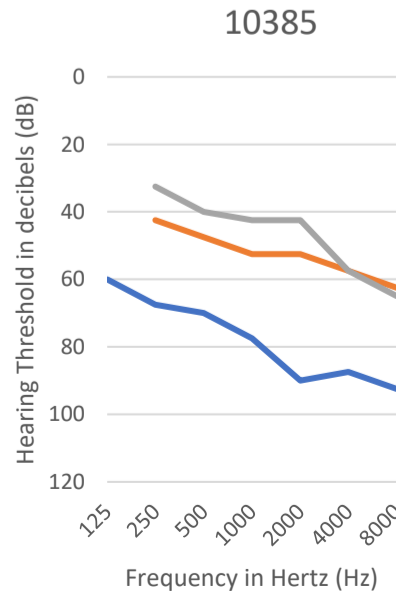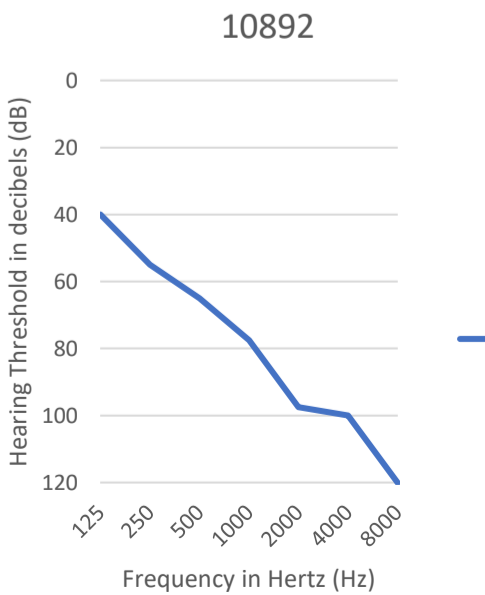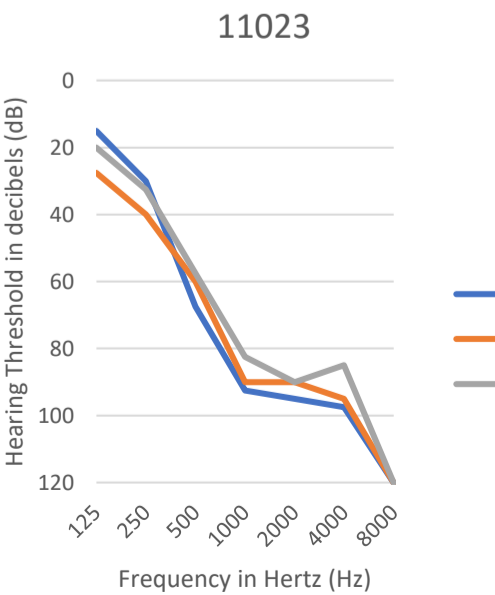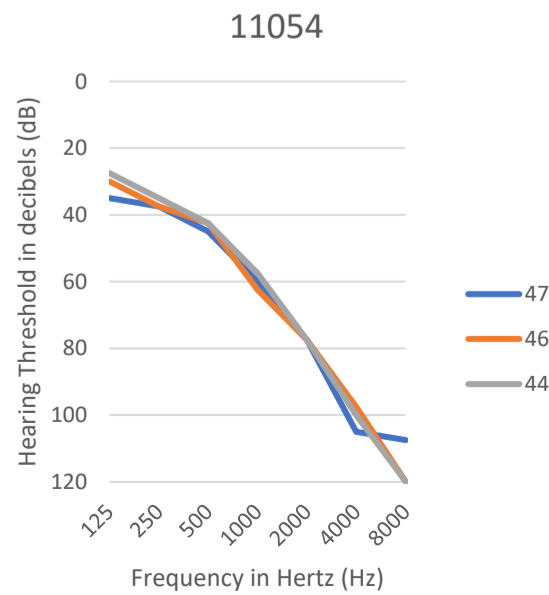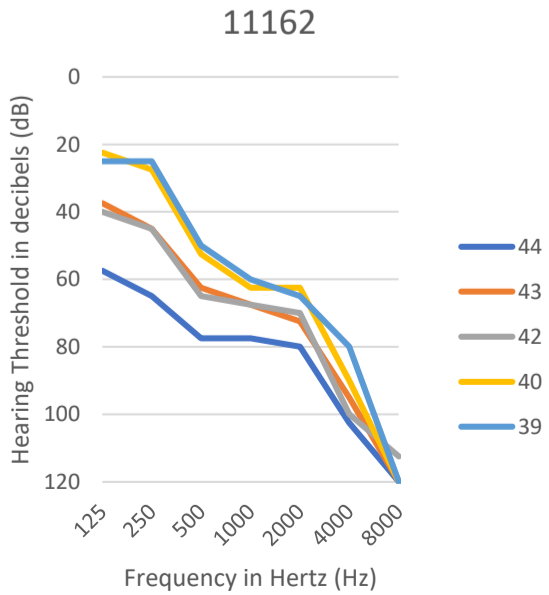

Supplement: Supplementary file 1 [file ijms-23-06029-s001.zip › Figure S1.pdf]
